# Neuroprotective and Anti-Inflammatory Effects of Kuwanon C from *Cudrania tricuspidata* Are Mediated by Heme Oxygenase-1 in HT22 Hippocampal Cells, RAW264.7 Macrophage, and BV2 Microglia

**DOI:** 10.3390/ijms21144839

**Published:** 2020-07-08

**Authors:** Wonmin Ko, Chi-Su Yoon, Kwan-Woo Kim, Hwan Lee, Nayeon Kim, Eun-Rhan Woo, Youn-Chul Kim, Dae Gill Kang, Ho Sub Lee, Hyuncheol Oh, Dong-Sung Lee

**Affiliations:** 1 College of Pharmacy, Chosun University, Dong-gu, Gwangju 61452, Korea; rabis815@naver.com (W.K.); ghksdldi123@hanmail.net (H.L.); rlaskdus1209@naver.com (N.K.); wooer@Chosun.ac.kr (E.-R.W.); 2 Institute of Pharmaceutical Research and Development, College of Pharmacy, Wonkwang University, Iksan 54538, Korea; ycs1991@naver.com (C.-S.Y.); swamp1@naver.com (K.-W.K.); yckim@wku.ac.kr (Y.-C.K.); hoh@wonkwang.ac.kr (H.O.); 3 Hanbang Cardio-Renal Syndrome Research Center, Wonkwang University, Iksan 54538, Korea; dgkang@wku.ac.kr (D.G.K.); host@wku.ac.kr (H.S.L.)

**Keywords:** heme oxygenase-1 regulation, *Curdrania tricuspidata*, kuwanon C, HT22 hippocampal cells, BV2 microglia, RAW264.7 macrophage

## Abstract

Heme oxygenase (HO)-1 is a detoxifying phase II enzyme that plays a role in both inflammatory and oxidative stress responses. *Curdrania tricuspidata* is widespread throughout East Asia and is used as a therapeutic agent in traditional medicine. We investigated whether treatment with sixteen flavonoid or xanthone compounds from *C. tricuspidata* could induce HO-1 expression in HT22 hippocampal cells, RAW264.7 macrophage, and BV2 microglia. In these compounds, kuwanon C showed the most remarkable HO-1 expression effects. In addition, treatment with kuwanon C reduced cytoplasmic nuclear erythroid 2-related factor (Nrf2) expression and increased Nrf2 expression in the nucleus. Significant inhibition of glutamate-induced oxidative injury and induction of reactive oxygen species (ROS) occurred when HT22 hippocampal cells were pretreated with kuwanon C. The levels of inflammatory mediator and cytokine, which increased following lipopolysaccharide (LPS) stimulation, were suppressed in RAW264.7 macrophage and BV2 microglia after kuwanon C pretreatment. Kuwanon C also attenuated p65 DNA binding and translocation into the nucleus in LPS-induced RAW264.7 and BV2 cells. The anti-inflammatory, anti-neuroinflammatory, and neuroprotective effects of kuwanon C were reversed when co-treatment with HO-1 inhibitor of tin protoporphyrin-IX (SnPP). These results suggest that the neuroprotective and anti-inflammatory effects of kuwanon C are regulated by HO-1 expression.

## 1. Introduction

Heme oxygenase (HO) catalyzes the degradation of heme and is found in three isoforms: HO-1, -2, and -3. HO-1 is a 32 kDa protein induced by diverse stimuli, including inflammatory cytokine expression and oxidative stress. HO-2 is a 36 kDa protein that commonly acts as an important constitutively expressed enzyme, and HO-3 is a 33 kDa protein important for heme sensing [1,2]. HO-1 is a detoxifying phase II enzyme that plays a critical role in both inflammatory and oxidative responses. HO-1-mediated degradation of heme generates carbon monoxide (CO), biliverdin, and iron, which are believed to facilitate several cytoprotective responses against oxidative stress [3]. Nuclear erythroid 2-related factor (Nrf2) regulates the expression of various cytoprotective genes, including *HO-1*. In addition, Nrf2 is known to serve an essential function in both the xenobiotic and oxidative stress responses, fulfilling a critical protective function in several organs, including the kidney, liver, nervous system, and lungs [4].

Neurodegenerative diseases such as Parkinson’s, Huntington’s, and Alzheimer’s disease, are characterized by dysfunction of the central nervous system (CNS) and progressive loss of neurons. Neuroinflammation and chronic oxidative stress are considered the main causes of these diseases, implying that HO-1 plays a key role in preventing pathogenesis through the regulation of inflammatory and oxidative stress responses [5].

*Curdrania tricuspidata* (Moraceae family) is widespread throughout East Asia and is a common ingredient in many traditional medicines. *C. tricuspidata* contains several classes of potentially therapeutic compounds, including xanthones, flavonoids, organic acids, and phenylpropanoids. Several studies have attempted to classify the abundant xanthones and flavonoids produced by these plants. In addition, *C. tricuspidata* is known to produce pharmacological activity, including anti-inflammatory, antioxidant, antitumor, hepatoprotective, neuroprotective, anti-obesity, antimicrobial, immunomodulatory, antiatherosclerotic, skin-protecting, and anti-diabetic effects [6]. In our previous study, we evaluated several *C. tricuspidata* compounds and their effects on HO-1 expression and reported that two compounds exert their biological effects through the regulation of HO-1 expression. Other studies have reported that cudratricusxanthone A exerts a protective effect on HT22 cells and has an anti-inflammatory effect in RAW264.7 cells [7,8]. In addition, cudraflavanones A have been shown to exert anti-neuroinflammatory effects in BV2 cells [9].

This study identifies new bioactive compounds from *C. tricuspidata* and describes their activity in various cell lines. In total, sixteen compounds were evaluated in neurodegenerative and inflammatory disease models. This study focused on identifying biomarkers that regulate the expression of HO-1 in three cell lines of neurodegenerative and inflammatory disease model: the immortalized murine hippocampal neuronal cell line HT22, the murine-derived macrophage cell line RAW264.7, and the immortalized murine microglia cell line BV2.

## 2. Results

### 2.1. Effects of Sixteen Compounds from C. tricuspidata on the Viability of HT22, RAW264.7, and BV2 Cells

**Figure 1:**
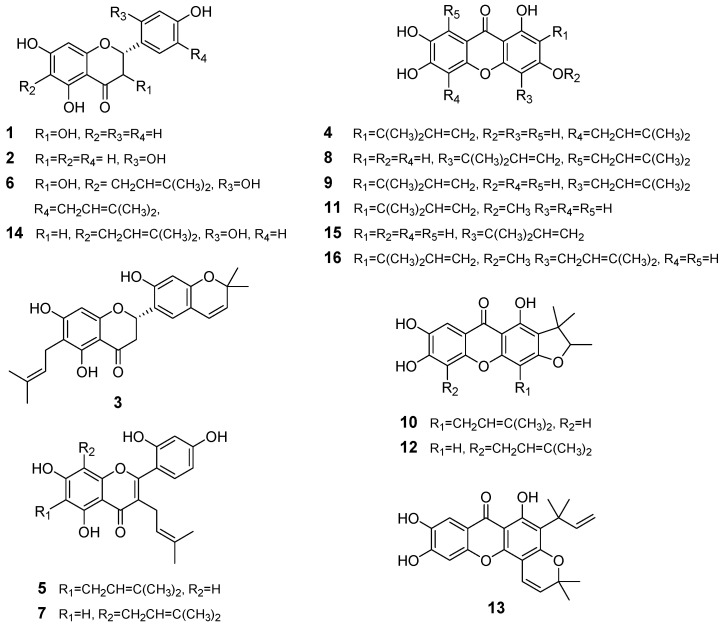
Chemical structures of the sixteen *C. tricuspidata* compounds used in this study.

The sixteen *C. tricuspidata* compounds used in this study were all isolated from the roots of the plant using our previously described isolation method. In addition, the chemical structures for these compounds, dihydrokaempferol (**1**), steppogenin (**2**), cudraflavanone A (**3**), cudraxanthone L (**4**), cudraflavone C (**5**), cudraflavanone D (**6**), kuwanon C (**7**), cudratricusxanthone A (**8**), macluraxanthone B (**9**), cudraxanthone M (**10**), 1,6,7-trihydroxy-2-(1,1-dimethyl-2-propenyl)-3-methoxyxanthone (**11**), cudraxanthone D (**12**), cudratricusxanthone N (**13**), cudraflavanone B (**14**), cudratricusxanthone L (**15**), and cudratricusxanthone A (**16**), have also been previously described [10]. To determine the cytotoxic effects of these compounds (CTC; *C. tricuspidata* compound, Figure 1), we treated HT22, RAW264.7, and BV2 cells with various concentrations of each compound for 24 h and subsequently performed an 3-(4,5-dimethylthiazol-2-yl)-2,5-diphenyltetrazolium bromide (MTT) assay (Supplementary Figures S1–S3). These MTT assays were then used to determine the highest nontoxic dosage of each compound, which was subsequently used in all experiments.

### 2.2. Effects of the Sixteen C. tricuspidata Compounds on HO-1 Protein Expression and Nuclear Translocation of Nrf2 in HT22, RAW264.7, and BV2 Cells

**Figure 2:**
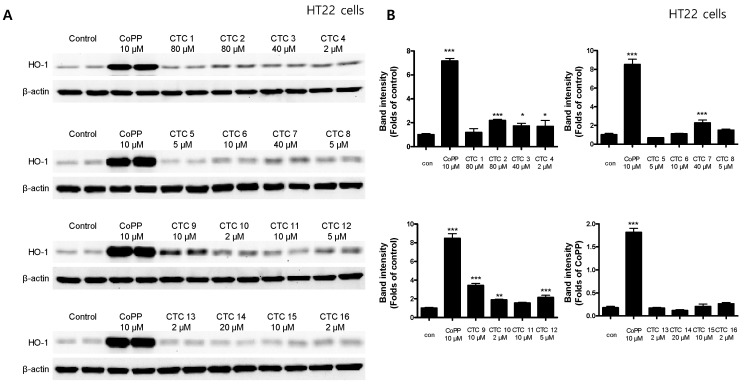
Effects of each of the sixteen *C. tricuspidata* compounds on the protein expression levels of HO-1 in HT22 cells. (**A**) Cells were treated for 12 h with the indicated concentration of each compound and assayed using Western blotting. Immunoblotting was performed using ImageJ software. (**B**) Band intensities were normalized to β-actin. CoPP (10 μM) was used as a positive control. Data are presented as the mean ± standard deviation of four independent experiments. * *p* < 0.05, ** *p* < 0.01, *** *p* < 0.001 when compared to the untreated control.

**Figure 3:**
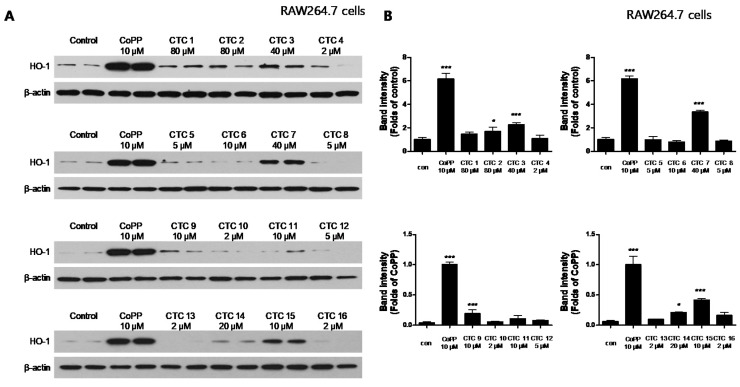
Effects of the sixteen *C. tricuspidata* compounds on the protein expression levels of HO-1 in RAW264.7 cells. (**A**) Cells were incubated for 12 h with the indicated concentration of each compound and analyzed using western blotting. (**B**) Band intensities were normalized to β-actin. CoPP (10 μM) was used as a positive control. Data are presented as the mean ± standard deviation of four independent experiments. * *p* < 0.05, *** *p* < 0.001 when compared to the control group.

To investigate whether any of these compounds induced the expression of HO-1 HT22, RAW264.7, and BV2 cells were treated with the indicated concentrations of each of the test compounds for 12 h and then assayed by Western blotting. Cobalt protoporphyrin (CoPP), an established HO-1 inducer, was used as a positive control. CTC **2**, **3**, **4**, **7**, **9**, **10**, and **12** significantly induced HO-1 expression in HT22 cells (Figure 2), whereas **3**, **7**, **9**, **14**, and **15** induced HO-1 expression in RAW264.7 cells (Figure 3) and CTC **1**, **2**, **3**, **7**, **9**, **10**, **11**, **12**, **13**, **14**, and **15**.

**Figure 4:**
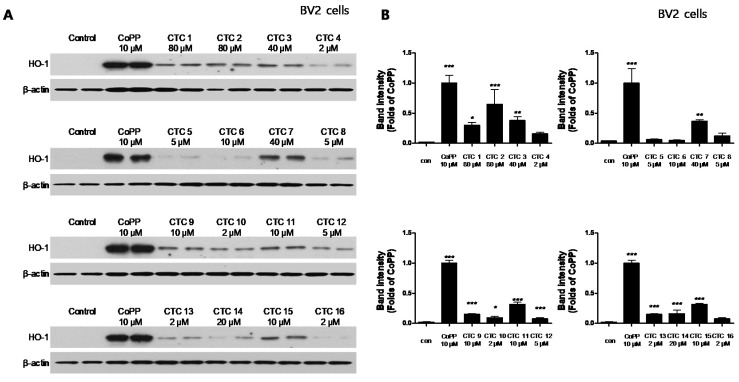
Effects of the sixteen *C. tricuspidata* compounds on the protein expression levels of HO-1 in BV2 cells. (**A**) Cells were incubated for 12 h with the indicated concentration of each compound and analyzed using western blotting. (**B**) Band intensities were normalized to β-actin. CoPP (10 μM) was used as a positive control. Data are presented as the mean ± standard deviation of four independent experiments. * *p* < 0.05, ** *p* < 0.01, *** *p* < 0.001 when compared to the control group.

**Figure 5:**
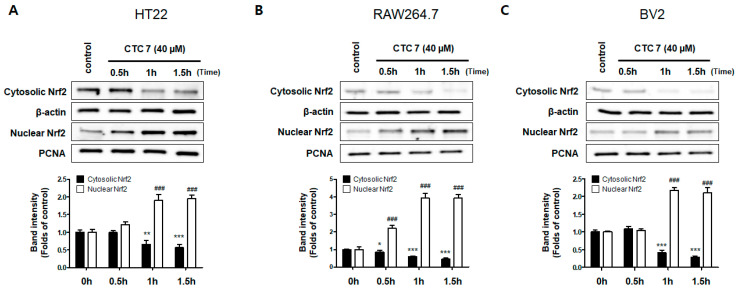
Effects of CTC **7** treatment on the nuclear translocation of nuclear factor erythroid-derived 2-related factor-2 (Nrf2) in HT22 (**A**), RAW264.7 (**B**), and BV2 (**C**) cells. These cells were treated with CTC **7** (40 μM) for 0.5, 1, or 1.5 h and then the nuclei were fractionated from the cytosol using a Caiman Nuclear Extraction Kit. Cytoplasmic and nuclear Nrf2 expression was then assayed using western blotting. Data represent the mean ± standard deviation for three independent experiments. * *p* < 0.05, ** *p* < 0.01 *** *p* < 0.001 when compared to the untreated cytosolic Nrf2 control. ### *p* < 0.001 when compared to the untreated nuclear Nrf2 control.

HO-1 expression in BV2 cells (Figure 4). CTC **3** and **7** were able to induce significant HO-1 expression in all three cell lines, with CTC **7** exhibiting the strongest induction. Thus, we examined the effect of CTC **7** treatment on the nuclear translocation of activated Nrf2. For this purpose, HT22, RAW264.7, and BV2 cells were treated with 40 μM CTC **7** for 0.5, 1, and 1.5 h, and Nrf2 expression was assayed using Western blotting. This assay showed that CTC **7** treatment induced the nuclear translocation of Nrf2 in a time-dependent manner (Figure 5).

### 2.3. Effect of CTC 7 Treatment on Glutamate-Induced Oxidative Injury and Reactive Oxygen Species (ROS) Generation in HT22 Cells

**Figure 6:**
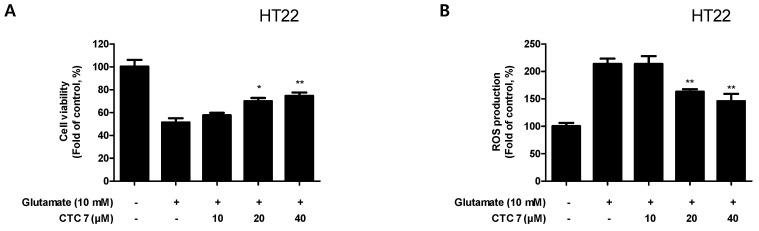
Effects of CTC **7** treatment on glutamate-induced (**A**) oxidative neurotoxicity and (**B**) ROS generation in HT22 cells. HT22 cells were pretreated with CTC **7** (10, 20, and 40 μM) for 3 h and then incubated with glutamate (10 mM) for 12 h. Data are presented as the mean ± standard deviation for three independent experiments. * *p* < 0.05, ** *p* < 0.01 when compared to glutamate-treated cells.

Based on the observation that CTC **7** treatment induced HO-1 protein expression in HT22, RAW264.7, and BV2 cells, we examined the neuroprotective capacity of CTC7 treatment, its anti-oxidative stress and anti-inflammatory effects, following lipopolysaccharide (LPS) challenge in HT22 cells. HT22 cells were pretreated with CTC **7** for 3 h and exposed to glutamate (10 mM) for 12 h. Pretreatment of HT22 cells with 20 or 40 μM of CTC **7** significantly reduced the effects of glutamate-induced oxidative injury (Figure 6A). In addition, pretreatment with 20 or 40 μM CTC **7** for 3 h also effectively decreased ROS production induced by glutamate treatment (Figure 6B).

### 2.4. Effects of CTC 7 Treatment on LPS-Induced Production of Nitric Oxide (NO), Prostaglandin E_2_ (PGE_2_), Interleukin (IL)-6, and Tumor Necrosis Factor (TNF)-α, and the Expression of Inducible Nitric Oxide Synthase (iNOS) and Cyclooxygenase (COX)-2 in RAW264.7 and BV2 Cells

**Figure 7:**
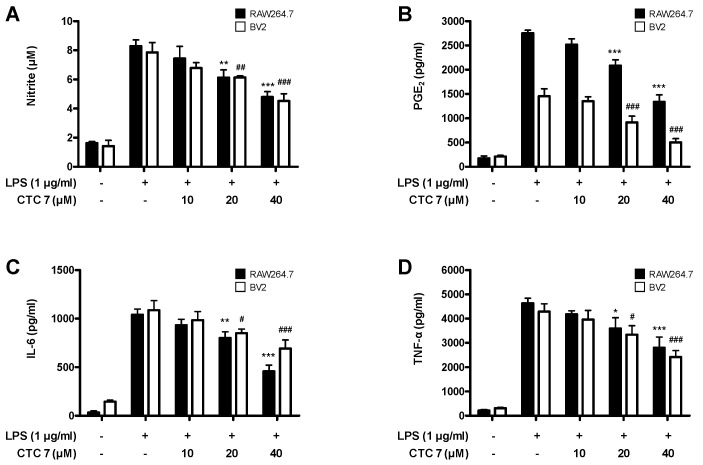
Effects of CTC **7** treatment on nitrite content (**A**) PGE_2_ (**B**), IL-6 (**C**), and TNF-α (**D**) expression in LPS-stimulated RAW264.7 and BV2 cells. Cells were pretreated for 3 h with the indicated concentrations of CTC **7** and stimulated for 24 h with LPS (1 μg/mL). Measurements of nitrite concentrations, a PGE_2_ assay, and an enzyme-linked immunosorbent assay (ELISA) were performed as described in the Materials and Methods. Bars represent the mean ± standard deviation for three independent experiments. * *p* < 0.05, ** *p* < 0.01, *** *p* < 0.001, # *p* < 0.05, ## *p* < 0.01, ### *p* < 0.001 compared with the LPS-treated group.

**Figure 8:**
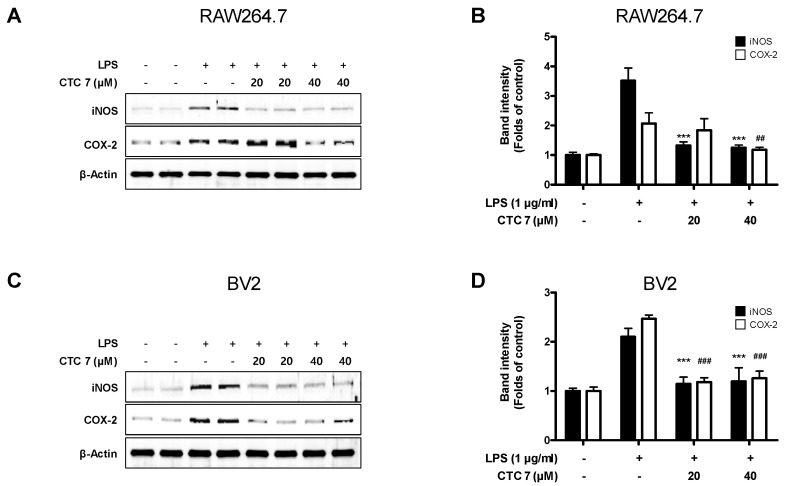
Effects of CTC **7** treatment on the protein expression levels of iNOS (**A**) and COX-2 (**B**) in LPS-stimulated RAW264.7 and BV2 cells. Cells were pretreated for 3 h with the indicated concentrations of CTC **7** and stimulated for 24 h with LPS (1 μg/mL). Western blot analysis was performed as described in the Materials and Methods. Immunoblotting was performed using ImageJ software. Band intensities were normalized to that of β-actin. *** *p* < 0.001, ## *p* < 0.01, ### *p* < 0.001 when compared with the LPS-treated control.

Given these results, we determined whether CTC **7** treatment could attenuate the overproduction of the proinflammatory mediators NO, PGE_2_, IL-6, and TNF-α in RAW264.7 and BV2 cells stimulated with LPS (1 μg/mL). The levels of NO, PGE_2_, IL-6, and TNF-α increased following LPS stimulation, and pretreatment with CTC **7** suppressed these responses in both RAW264.7 and BV2 cells (Figure 7). Furthermore, the expression of iNOS and COX-2 proteins increased in LPS-treated groups, and these responses were also repressed in cells pretreated with CTC **7** (Figure 8).

### 2.5. Effect of CTC 7 Treatment on LPS-Induced Activation of Nuclear Factor Kappa B (NF-κB) Signaling in RAW264.7 and BV2 Cells

**Figure 9:**
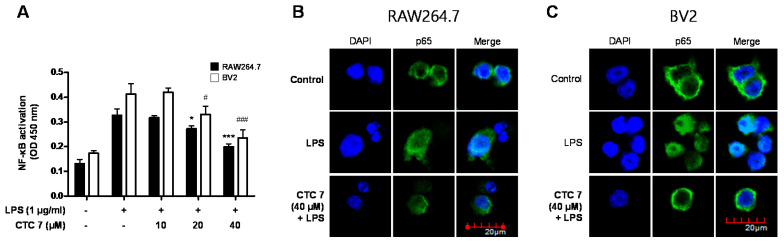
Effects of CTC **7** treatment on NF-κB DNA-binding activity (**A**) and NF-κB localization (**B**,**C**) in RAW264.7 and BV2 cells. Cells were pretreated with the indicated concentrations of CTC **7** for 3 h and stimulated with LPS (1 μg/mL) for 1 h. A commercially available NF-κB ELISA kit was used to test nuclear extracts and determine the level of NF-κB binding in each of the samples. Data represent the mean values of three independent experiments. * *p* < 0.05, *** *p* < 0.001, # *p* < 0.05, ### *p* < 0.001 when compared to the LPS-treated control.

Activation of the NF-κB signaling pathway is required for LPS-induced production of proinflammatory mediators [11]. Therefore, we investigated whether CTC **7** treatment affects the activation of the NF-κB signaling pathway in LPS-induced RAW264.7 and BV2 cells. Cells were pretreated with CTC **7** for 3 h and stimulated with LPS for 1 h. The DNA binding activity of p65 was shown to increase the response to LPS stimulation, and pretreatment with CTC **7** attenuated this DNA binding activity in both RAW264.7 and BV2 cells (Figure 9A). In addition, immunofluorescence analysis showed that the p65 subunit was translocated into the nucleus following LPS stimulation and that pretreatment with CTC **7** blocked this response in both RAW264.7 (Figure 9B) and BV2 cells (Figure 9C).

### 2.6. Effect of CTC 7 Treatment on LPS-Induced NF-κB Activation in RAW264.7 and BV2 Cells

**Figure 10:**
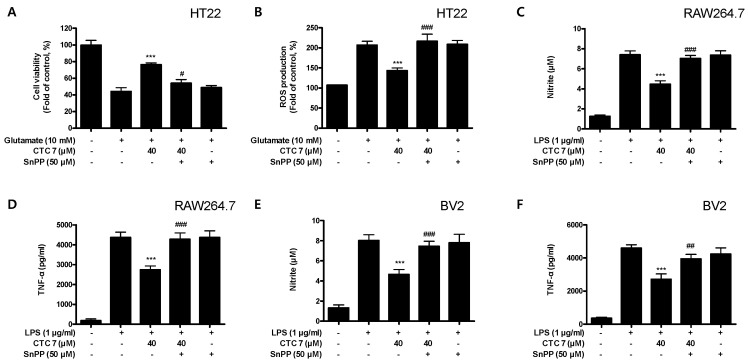
Effects of CTC **7**-induced Nrf2-mediated HO-1 expression on glutamate-induced oxidative neurotoxicity and ROS generation in HT22 (**A**,**B**) cells and LPS-stimulated proinflammatory mediator production in RAW264.7 (**C**,**D**) and BV2 (**E**,**F**) cells. HT22 cells were treated with CTC **7** (40 μM) in the presence or absence of SnPP (50 μM). (**A**) Cell viability and (**B**) ROS generation were measured following treatment with glutamate (10 mM) for 12 h. RAW264.7 and BV2 cells were pretreated with CTC **7** (40 μM) for 3 h with or without SnPP (50 μM), and subsequently stimulated with LPS (1 μg/mL) for 24 h. Nitrite (**C**,**E**) and TNF-α (**D**,**F**) were assayed as described in the Materials and Methods section. Data are presented as the mean ± standard deviation for three independent experiments. *** *p* < 0.001 compared to the glutamate or LPS control; # *p* < 0.05, ## *p* < 0.01, ### *p* < 0.001 compared to CTC7 plus glutamate or LPS without SnPP.

To determine whether the neuroprotective and anti-inflammatory effects of CTC **7** are correlated with HO-1 expression in HT22, RAW264.7, and BV2 cells, we included a set of experiments with tin protoporphyrin-IX (SnPP), which is a selective HO-1 inhibitor. After the cells were treated with 40 μM CTC **7** for 3 h with or without 50 μM SnPP, the cells were treated with glutamate for another 12 h (HT22) or LPS for 24 h (RAW264.7 and BV2 cells) and assayed by ELISA. Pretreatment with CTC **7** protected HT22 cells against glutamate-induced oxidative injury (Figure 10A) by reducing ROS production in these cells (Figure 10B). However, these responses were effectively eliminated by the simultaneous addition of SnPP (Figure 10A,B). SnPP did not affect cell viability or ROS production in glutamate-treated HT22 cells, suggesting that these changes were the result of the direct inhibitory effect of SnPP on HO-1. In addition, pretreatment with CTC **7** resulted in decreased nitrite and TNF-α production in LPS-induced RAW264.7 (Figure 10C,D) and BV2 (Figure 10E,F). However, the anti-inflammatory effects of CTC **7** were also reversed by SnPP in both cell types. Similar to HT22 cells, SnPP alone did not affect NO or TNF-α production following LPS stimulation, which suggests that the neuroprotective and anti-inflammatory effects of CTC **7** treatment are regulated by HO-1 expression.

## 3. Discussion

HO-1 enzymes have been used as important markers in the regulation of various diseases, including obesity and vascular diseases [12], kidney injury [13], diabetes [14], and digestive disorders [15]. HO-1 expression has also been linked to neuroprotective and neurotoxic effects, and it is upregulated in brain tissues following various oxidative stress-inducing pathological stimuli [5,16]. In addition, it has been reported that CO production through heme degradation by HO-1 exerts anti-inflammatory effects [17]. Many studies have shown that various types of natural products regulate HO-1 expression, which we confirmed in a recent study, suggesting that one of the pathways for its regulation could be the Nrf-2 pathway [18,19,20]. Accordingly, this study focused on the discovery and validation of natural products that exert a regulatory effect on inflammatory and degenerative diseases via changes in HO-1 expression. First, we confirmed the cytotoxic effects of the sixteen compounds (Supplementary Figures S1–S3) and evaluated their effects on HO-1 expression in HT22, BV2, and RAW264.7 cells. A total of seven compounds (Steppogenin, cudraflavanone A, cudraxanthone L, kuwanon C, macluraxanthone B, cudraxanthone M, and cudraxanthone D) in HT22 (Figure 2), five compounds (steppogenin, cudraflavanone A, kuwanon C, macluraxanthone B, and 16,7-trichydroxy-2-(1,1-dimethyl-2-propenyl)-3-methyloxanthone) in RAW264.7 cells (Figure 3), and nine compounds (dihydrokaempferol, steppogenin, cudraflavanone A, kuwanon C, macluraxanthone B, cudraxanthone M, 1,6,7-trihydroxy-2-(1,1-dimethyl-2-propenyl)-3-methoxyxanthone, cudraxanthone D, and cudraflavanone B) in BV2 cells (Figure 4) were shown to significantly upregulate HO-1 expression. Of these, four compounds—steppogenin, cudraflavanone A, kuwanon C, and macluraxanthone B—were shown to affect HO-1 expression in all three cell lines including HT22, BV2, and RAW264.7.

Steppogenin of flavanone skeletons has been studied for its cyclooxygenase [21] and tyrosinase inhibitory effects [22,23,24,25,26]. In our previous study, we showed that steppogenin altered the anti-neuroinflammatory response via NF-kB and mitogen-activated protein kinase (MAPK) signaling suppression in microglia [27]. Cudraflavanone A, a prenylated flavanone, has been reported to inhibit topoisomerase I and protein kinase C (PKC) activity, leading to the induction of apoptotic cell death in human cancer cells [28] and vascular smooth muscle cell growth via a protein kinase B (Akt)-dependent pathway [29]. Our previous study also reported the anti-neuroinflammatory effects of cudraflavanone A via induction of HO-1 expression [9]. Macluraxanthone B, a prenylated xanthone, has been shown to exert anti-human immunodeficiency virus (HIV) [30], hepatoprotective [31], cytotoxic [32], and antitumor effects [33]. Kuwanon C, a prenylated flavone, has been shown to exert a number of biological effects, including an inhibitory effect on platelet aggregation induced by platelet-activating factor (PAF) and arachidonic acid [34], mild cyclooxygenase inhibition, strong lipoxysgene inhibition [35], tyrosinase inhibition [36,37], anti-microbial effects [38], melanin inhibition [39], cytotoxic effects [40,41], cholinesterase inhibition [42], skin whitening effects [43], anti-obesity effects [44], anti-inflammatory effects on only reducing of NO production [44,45,46], antioxidant effects [47], mild cathepsin K inhibition [48], pancreatic lipase inhibition [49], protective effects in a spinal cord injury model [50], antiproliferative effects [51], and attenuation of MPTP-induced Parkinson’s disease [52]. As a result of this study, among the sixteen compounds, kuwanon C showed the most remarkable effect for inducing HO-1 in all of three cells, and this is first report to biological action of kuwanon C though the HO-1 regulation in the HT22 hippocampal, BV2 microglia, and RAW264.7 macrophage cells. Therefore, we conducted further study of kuwanon C in three cell lines.

The Nrf2 transcription factor plays an important role in antioxidant responsive element (ARE)-mediated expression of phase 2 detoxifying genes and in the activation of other inducible genes, including HO-1 [53]. A previous study showed that the induction of HO-1 is primarily regulated at the transcriptional level, and its induction is often related to Nrf2 activation. Therefore, regulating the degree of oxidative stress via upregulation of Nrf2-mediated HO-1 may be an important factor in developing a treatment strategy for neurological diseases [54]. As a result, we confirmed that kuwanon C treatment induces the translocation of Nrf2 to the nucleus in HT22, BV2, and RAW264.7 cell lines (Figure 5). Neuronal oxidative stress has been postulated to be the underlying basis for neuronal cell death in neurodegenerative diseases [55,56], and HT22 hippocampal cells have played a critical role in developing neurodegenerative disease models. Glutamate is known to generate oxidative stress, leading to the depletion of glutathione, elevated Ca^2+^ levels, and increased ROS production [57]; however, HT22 cells lack functional ionotropic glutamate receptors and cause glutamate-mediated HT22 cell death [58]. Here, we demonstrated that kuwanon C treatment generates an effective antioxidant effect that protects against glutamate-induced ROS production, cellular damage, and cell death (Figure 6), confirming that this compound may have some value in the treatment of neurodegenerative diseases.

The initiating pathoetiology of neurodegenerative disorders involves an inflammatory process in response to a diverse array of CNS stimuli [59], and microglia, which are involved in the neuroinflammation process, are directly related to cognitive impairment and the neurodegenerative process [60]. Inflammation is a vital component of the immune response that protects against damage and infection. However, excessive acute or chronic inflammation can result in serious pathologies, including arthritis, asthma, inflammatory bowel disease, neurodegenerative disorders, and sepsis [61]. Macrophages play a prominent role in inflammatory diseases and produce a variety of inflammatory cytokines, including TNF-α, IL-1β, IL-6, and other inflammatory mediators induced by LPS. Macrophages also produce proinflammatory and cytotoxic mediators, including NO and prostaglandins (PGs), through the activity of their inducible enzymes, such as iNOS and COX-2 [61,62]. In macrophages, the NF-κB pathway is a master regulator of the molecular inflammatory response and is involved in the expression and production of proinflammatory mediators and cytokines. Under normal conditions, NF-κB is made up of inactive p50 and p65 subunits bound to the inhibitor of NF-κB (IκB-α), but, when this pathway is activated by LPS or other stimuli, it phosphorylates IκB-α, leading to its degradation and the subsequent translocation of NF-κB into the nucleus [63]. Macrophage-like RAW264.7 and microglia-like BV2 cells are useful for evaluating these anti-inflammatory effects. In this study, we showed that kuwanon C effectively inhibited NO, PGE_2_, IL-6, and TNF-α production in BV2 and RAW264.7 cells (Figure 7). In addition, iNOS and COX-2 were also effectively inhibited by this compound (Figure 8), and NF-κB activation and its translocation into the nucleus were also inhibited by kuwanon C treatment (Figure 9). Because HO-1 and Nrf2 are known to be critical to the regulation of oxidative damage and inflammation in most cell types [4], we examined whether antioxidative and anti-inflammatory effects were directly related to the modification of their expression by testing the effects of kuwanon C in the presence of an HO-1 inhibitor. Using this approach, we confirmed that the antioxidative and anti-inflammatory effects of kuwanon C were mediated by HO-1 and Nrf2 in HT22, BV2, and RAW264.7 cells (Figure 10). These results suggest that HO-1 mediates the effects of kuwanon C and that these effects are regulated by the Nrf2 pathway.

The focus of this study is to find new activity of sixteen compounds isolated from *C. tricuspidata* through the HO-1 regulatory action in three different cell lines including HT22, BV2, and RAW264.7. We find that kuwanon C is shown to be an effective HO-1 inducer in three cell lines, which is critical for understanding inflammatory and oxidative stress responses. Therefore, this study has been meaningful as the first report that among the sixteen compounds kuwanon C was also shown to exert neuroprotective effects in HT22 cells, anti-inflammatory effects in RAW264.7, and anti-neuroinflammatory effects in BV2 cells via Nrf2-mediated HO-1 regulation. These results suggest that the mechanism of HO-1 regulation could be important in identifying neuroprotective or anti-inflammatory compounds derived from natural products, and may provide insights into future intervention strategies to treat neurodegenerative or inflammatory diseases. Based on the present study, the further studies are needed to use *C. tricuspidata* extract or kuwanon C for the commercial or wide uses. Finally, we think that *C. tricuspidata* extract or kuwanon C might be an advantageous candidate for the treatment of neurodegenerative or inflammatory diseases.

## 4. Materials and Methods

### 4.1. Materials

Tissue culture reagents, such as Roswell Park Memorial Institute 1640 (RPMI 1640), Dulbecco’s Modified Eagle’s Medium (DMEM), and fetal bovine serum (FBS), were purchased from Gibco BRL Co. (Grand Island, NY, USA). All chemicals were obtained from Sigma-Aldrich Chemical Co. (St. Louis, MO, USA). Primary antibodies, including anti-HO-1, anti-Nrf2, anti-p65, anti-COX-2, anti-iNOS, anti-β-actin, and anti-proliferating cell nuclear antigen (PCNA) were purchased from Santa Cruz Biotechnology (Santa Cruz, CA, USA), and anti-rabbit and anti-mouse secondary antibodies were purchased from Millipore (Billerica, MA, USA). ELISA kits for PGE_2_, IL-6, and TNF-α were purchased from R&D Systems, Inc. (Minneapolis, MN, USA). The structure of each of the sixteen *C. tricuspidata* compounds was determined by analyzing various spectroscopic data, including mass spectroscopy and nuclear magnetic resonance (NMR) data, which were consistent with what had been previously reported [10].

### 4.2. Cell Culture and Viability Assays

HT22 and BV2 cells were donated by Prof. Youn-Chul Kim (College of Pharmacy, Wonkwang University, Iksan, Republic of Korea). RAW264.7 was purchased from American Type Culture Collection (ATCC, Manassas, VA, USA). HT22 cells were cultured in DMEM supplemented with 1% penicillin–streptomycin and 10% heat-inactivated FBS at 37 °C in a humidified 5% CO_2_ and 95% air atmosphere. RAW264.7 and BV2 cells were maintained at 5 × 10^5^ cells/mL in RPMI 1640 supplemented with 10% heat-inactivated fetal bovine serum, l-glutamine (2 mM), penicillin G (100 units/mL), and streptomycin (100 mg/mL). The cell culture and viability assays were conducted according to a previously described method [20,64].

### 4.3. Western Blot Analysis and Extraction of Cytoplasmic and Nuclear Cell Fractions

The pelleted HT22, RAW264.7, and BV2 cells were washed with phosphate-buffered saline (PBS) and subsequently lysed in radioimmunoprecipitation assay (RIPA) buffer. The cytoplasmic and nuclear fractions were extracted using the Caiman Nuclear Extraction Kit (Cayman, Ann Arbor, MI, USA), and each fraction was lysed according to the manufacturer’s instructions. The detailed procedures for Western blot analysis were described in our previous report [64].

### 4.4. Reactive Oxygen Species Generation Assays

To evaluate ROS generation, HT22 cells were incubated with glutamate (10 mM) with or without CTC **7** (10–40 μM) and SnPP (HO inhibitor) (50 μM). Treated cells were then incubated at 37 °C for 12 h after glutamate treatment, washed and treated with Hank’s balanced salt solution containing 10 μM 2′,7′-dichlorofluorescein diacetate (DCFDA), and placed in the dark for 60 min. Then, the medium was removed, the cells were washed twice, and extracted with 1% Triton X-100 in PBS at 37 °C for 10 min. The fluorescence of each sample was evaluated at 490 nm and 525 nm using a SpectraMax Gemini XS (Molecular Devices, Sunnyvale, CA, USA).

### 4.5. Determination of Nitrite Concentrations

Nitrite, a stable end product of NO oxidation, was used as an indicator of NO production in each cell type, as previously described. [64,65].

### 4.6. PGE_2_ Assay

RAW264.7 and BV2 cells were cultured in 48-well plates, pretreated with different concentrations of CTC **7** for 3 h, and then stimulated with LPS (1 μg/mL) for 24 h. The culture media were collected to determine PGE_2_ level present in each sample using a commercially available kit from R&D Systems, Inc. (Minneapolis, MN, USA). Three independent assays were performed according to the manufacturer’s instruction.

### 4.7. Assays for IL-6 and TNF-α

Culture medium was collected from each of the treatment groups and used to determine IL-6 and TNF-α expression using ELISA kits tailored to each collection process (R&D Systems, Inc., Minneapolis, MN, USA), according to the manufacturer’s instructions. Briefly, RAW264.7 and BV2 cells were seeded in 48-well culture plates at a density of 5 × 10^5^ cells/well. After incubation, the supernatant was collected and used in the cytokine ELISA kits to measure the concentrations of IL-6 and TNF-α.

### 4.8. NF-κB Localization and Immunofluorescence

To study the localization of NF-κB, RAW264.7 and BV2 cells were grown on Lab-Tek II chamber slides and treated with 40-μM CTC **7** for 3 h before LPS stimulation (1 μg/mL) for 1 h. Cells were then fixed in formalin and permeabilized using cold acetone. The detailed procedures were described in our previous report [66].

### 4.9. Statistical Analysis

Data are presented as the mean ± standard deviation for three or four independent experiments. One-way analysis of variance, followed by Tukey’s multiple comparison tests, was used to compare the three groups. All statistical analyses were performed using GraphPad Prism software, version 5.01 (GraphPad Software Inc., San Diego, CA, USA).

## 5. Conclusions

Analysis of the bioactivity of sixteen compounds from the roots of *C. tricuspidata* revealed that the flavonoid compound kuwanon C exerts antioxidant effects in hippocampal cell lines and anti-inflammatory effects in both macrophage and microglial cell lines. In addition, this study demonstrates that the expression of HO-1 modulates cellular responses through the Nrf2 pathway. This study could provide the basis for clinical application of kuwanon C in the prevention and mitigation of oxidative injury.

## Supplementary Materials

The following are available online at https://www.mdpi.com/1422-0067/21/14/4839/s1. Figure S1. Cytotoxicity of CTC 1-16 on HT22 cells. HT22 cells were treated with the various concentration of CTC 1–16 for 48 h. The viability of cells subjected to different concentrations of CTC 1-16 was determined by MTT assay. Data are presented as the mean standard deviation of three independent experiments. * *p* < 0.05, ** *p* < 0.01 vs. non-treated control. Figure S2. Cytotoxicity of CTC 1-16 on RAW264.7 cells. RAW264.7 cells were treated with the various concentration of CTC 1–16 for 48 h. The viability of cells subjected to different concentrations of CTC 1-16 was determined by MTT assay. Data are presented as the mean standard deviation of three independent experiments. * *p* < 0.05, ** *p* < 0.01, *** *p* < 0.001 vs. non-treated control. Figure S3. Cytotoxicity of CTC 1-16 on BV2 cells. BV2 cells were treated with the various concentration of CTC 1–16 for 48 h. The viability of cells subjected to different concentrations of CTC 1-16 was determined by MTT assay. Data are presented as the mean standard deviation of three independent experiments. ** *p* < 0.01, *** *p* < 0.001 vs. non-treated control.
