# Supplementary material for: Neuroprotective and Anti-Inflammatory Effects of Kuwanon C from Cudrania tricuspidata Are Mediated by Heme Oxygenase-1 in HT22 Hippocampal Cells, RAW264.7 Macrophage, and BV2 Microglia
Source: Int J Mol Sci. 2020 Jul 8;21(14):4839. doi: 10.3390/ijms21144839 (PMC7402286; doi:10.3390/ijms21144839)
Supplement: Supplementary file 1 [file ijms-21-04839-s001.pdf]

Supplementary Materials

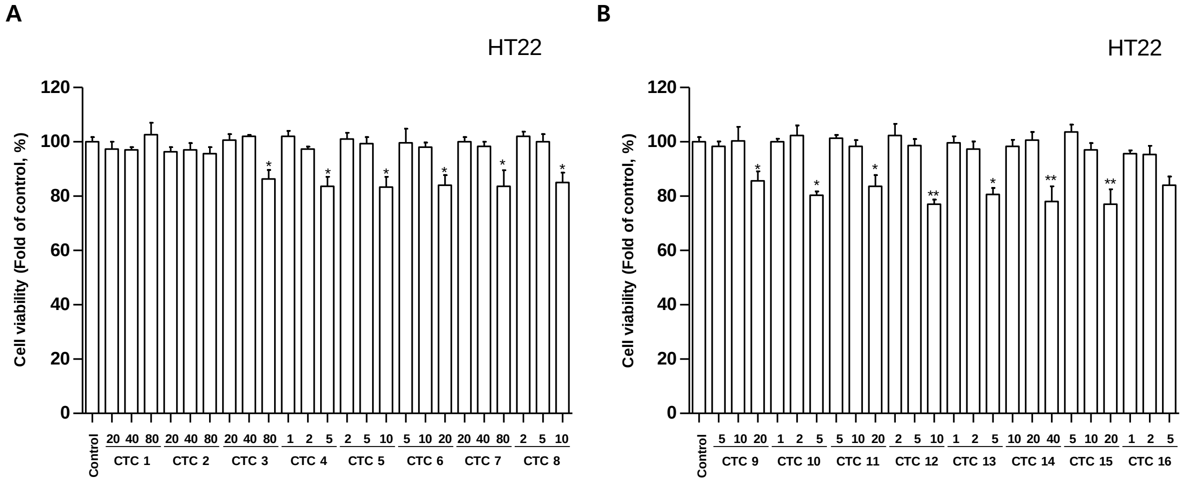

Figure S1. Cytotoxicity of CTC **1**-**16** on HT22 cells. HT22 cells were treated with the various concentration of CTC **1**–**16** for 48 h. The viability of cells subjected to different concentrations of CTC **1**-**16** was determined by MTT assay. Data are presented as the mean standard deviation of three independent experiments. **p* < 0.05, ***p* < 0.01 vs. non-treated control.

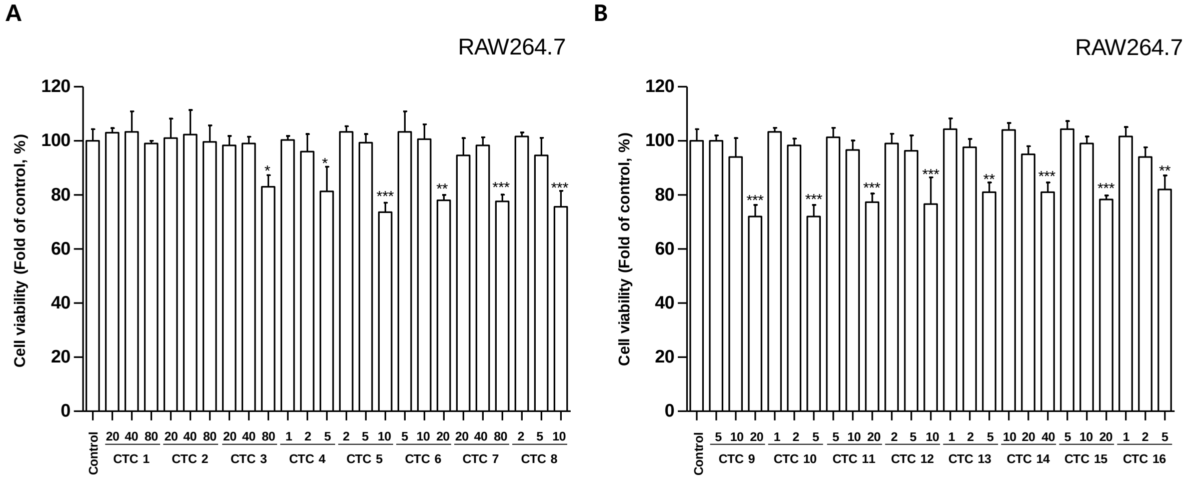

Figure S2. Cytotoxicity of CTC **1**-**16** on RAW264.7 cells. RAW264.7 cells were treated with the various concentration of CTC **1**–**16** for 48 h. The viability of cells subjected to different concentrations of CTC **1**-**16** was determined by MTT assay. Data are presented as the mean standard deviation of three independent experiments. **p* < 0.05, ***p* < 0.01, ****p* < 0.001 vs. non-treated control.

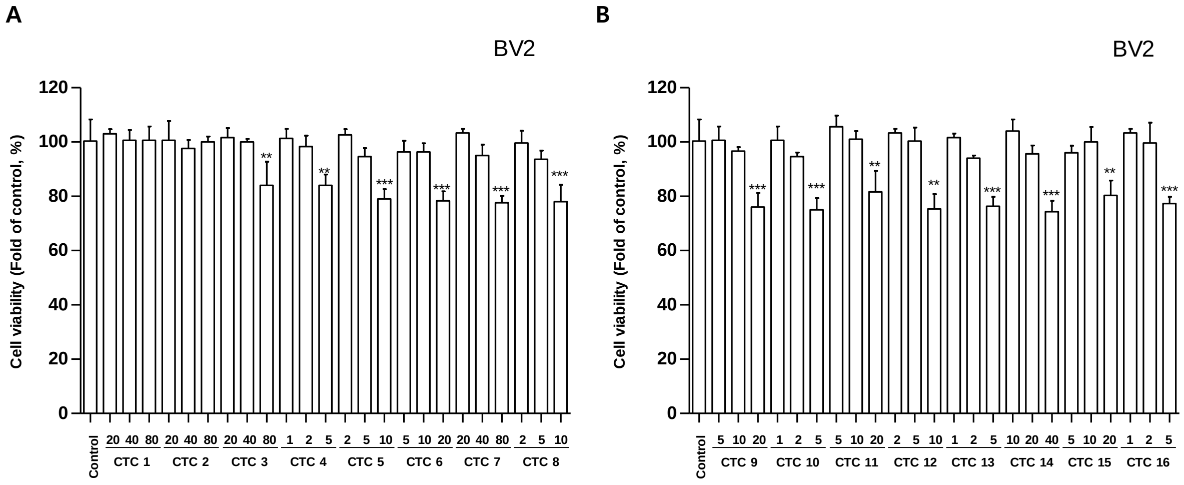

Figure S3. Cytotoxicity of CTC **1**-**16** on BV2 cells. BV2 cells were treated with the various concentration of CTC **1**–**16** for 48 h. The viability of cells subjected to different concentrations of CTC **1**-**16** was determined by MTT assay. Data are presented as the mean standard deviation of three independent experiments. **$p < 0.01$, ***$p < 0.001$ vs. non-treated control.
